# Polymer Nano‐Carrier‐Mediated Gene Delivery: Visualizing and Quantifying DNA Encapsulation Using dSTORM

**DOI:** 10.1002/smll.202405929

**Published:** 2024-11-17

**Authors:** Xhorxhina Shaulli, Aura Maria Moreno‐Echeverri, Mariza Andoni, Eileen Waeber, Shivaprakash N. Ramakrishna, Cornelia Fritsch, Dimitri Vanhecke, Barbara Rothen‐Rutishauser, Frank Scheffold

**Affiliations:** ^1^ Department of Physics University of Fribourg Chemin du Musée 3 Fribourg CH 1700 Switzerland; ^2^ Adolphe Merkle Institute University of Fribourg Chemin des Verdiers 4 Fribourg CH 1700 Switzerland; ^3^ Department of Materials ETH Zürich Vladimir‐Prelog‐Weg 1‐5/10 Zürich CH 8093 Switzerland; ^4^ Department of Biology University of Fribourg Chemin du Musée 10 Fribourg CH 1700 Switzerland

**Keywords:** cellular uptake, DNA encapsulation, drug delivery, dSTORM, microgels, polyplex systems, super‐resolution microscopy

## Abstract

The success of gene therapy hinges on the effective encapsulation, protection, and compression of genes. These processes deliver therapeutic genes into designated cells for genetic repair, cellular behavior modification, or therapeutic effect induction. However, quantifying the encapsulation efficiency of small molecules of interest like DNA or RNA into delivery carriers remains challenging. This work shows how super‐resolution microscopy, specifically direct stochastic optical reconstruction microscopy (dSTORM), can be employed to visualize and measure the quantity of DNA entering a single carrier. Utilizing pNIPAM/bPEI microgels as model nano‐carriers to form polyplexes, DNA entry into the carrier is revealed across different charge ratios at temperatures below and above the volume phase transition of the microgel core. The encapsulation efficiency also depends on DNA length and shape. This work demonstrates the uptake of the carrier entity by primary derived macro‐phages and showcases the cell viability of the polyplexes. The study shows that dSTORM is a potent tool for fine‐tuning and creating polyplex microgel carrier systems with precise size, shape, and loading capacity at the individual particle level. This advancement shall contribute significantly to optimizing gene delivery systems.

## Graphical abstract

This study demonstrates the use of super‐resolution microscopy (dSTORM) to quantify DNA encapsulation within pNIPAM/bPEI microgels. By visualizing DNA encapsulation at various charge ratios and for different temperatures, insights are provided into gene delivery carrier design, highlighting the impact of DNA size, shape, and microgel properties on encapsulation efficiency.

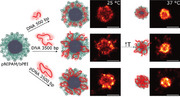

## 1 Introduction

The use of non‐viral vectors as carriers of genetic material for addressing various diseases has now proven successful and several approved drugs are already on the market.^[^ ^1^, ^2^ ^]^ This includes lipid‐based mRNA vaccines for COVID‐19,^[^ ^3^ ^]^ opening avenues for innovative developments in the field of gene therapy.^[^ ^4^ ^]^ Non‐viral vectors show promise due to their potentially low cytotoxicity and immunogenicity, structural diversity, ease of chemical and bio‐functionalization potential for large‐scale production, and affordability. Lipids,^[^ ^5^, ^6^, ^7^ ^]^ polymers,^[^ ^8^, ^9^, ^10^, ^11^, ^12^, ^13^, ^14^ ^]^ organic–inorganic hybrids^[^ ^15^ ^]^ and exosomes^[^ ^16^ ^]^ present some of the non‐viral carriers considered for gene delivery. Colloidal microgels represent a particularly interesting class of polymeric systems utilized as drug delivery vehicles.^[^ ^17^, ^18^, ^19^ ^]^ These are cross‐linked polymer particles with a size in the range of several hundred nanometers. Microgels offer unique advantages for polymer‐based drug delivery systems. Hybrid microgels with a cationic polymer shell are candidates for gene delivery applications due to their porous structure, which facilitates the incorporation of large molecules.^[^ ^20^ ^]^ They are often temperature‐tunable and exhibit a volume phase transition (VPT) around body temperature. When polymers complex with nucleic acids (NAs) like DNA or RNA, they form so‐called polyplexes where the polycationic shell masks the negative charges of the DNA.

For a polyplex to be considered a suitable carrier for NAs it must, among others, be capable of compacting, encapsulating, and protecting the genetic material (NAs).^[^ ^21^, ^22^ ^]^ Considering the numerous challenges these complexes must overcome, the demands on the vectors' performance are high and multifaceted. Indeed, many non‐viral formulations that have not yet reached clinical trials still suffer from either the loading capacity, high polydispersity, stability, or low reproducibility.^[^ ^23^ ^]^ These shortcomings are attributed to the sensitivity of complex formation to various experimental conditions, as highlighted in a recent review. The authors also proposed pathways for establishing standards for documenting polyplex formation.^[^ ^24^ ^]^ While the number of DNA molecules per particle is crucial for optimizing therapeutic formulations, accurately determining this quantity using conventional methods can be challenging.^[^ ^25^ ^]^ Quantifying DNA can enhance the precision, efficiency, and efficacy of delivery, while also aiding in the management of consistency and reproducibility of the carrier system in clinical trials.

In the present work, we study poly(N‐isopropyl‐acrylamide)/branched polyethylenimine (pNIPAM/bPEI) microgel polyplexes encapsulating different types of DNA, including shorter, longer, and plasmid DNA. Our approach utilizes direct stochastic optical reconstruction microscopy (dSTORM), offering precise insights into DNA quantities at near‐physiological temperatures. Generally, the pNIPAM microgel core helps to produce more monodisperse particles, which is impossible to achieve with bPEI alone. Additionally, the microgel swelling increases carrier porosity, facilitating DNA uptake while tightly enclosing the cargo at body temperatures, which are well above the volume phase transition temperature of 33 °C, where the microgel core collapses. We compare the encapsulation efficiency of short linear DNA (DNA 500 bp), long linear DNA (DNA 3527 bp), and plasmid DNA (pDNA 3527 bp) in polyplex systems.

As a drug delivery system, we have chosen pNIPAM/bPEI core‐shell microgels with a thermoresponsive pNIPAM core and a positively charged bPEI shell, a known subclass of stimuli‐responsive materials.^[^ ^11^, ^26^, ^27^, ^28^, ^29^ ^]^ with a size (diameter) of approximately 800 nm at room temperature. pNIPAM microgels are thermoresponsive, capable of adjusting their size and density profile based on temperature. Above the lower critical solution temperature (*T*_LCST_ ∼33 °C), the microgel's polymer network collapses, leading to the expulsion of water through a reversible process called a volume phase transition. Conversely, below 33 °C, the microgel's network swells. The microgels expand to typically to double their original size and thus consist of more than 80% water that can be partly replaced by the carrier material. Microgels have been extensively studied and considered for a variety of applications. For a comprehensive overview, we refer to the literature.^[^ ^30^, ^31^, ^32^, ^33^ ^]^

Our aim is to establish a quantitative method for assessing the amount of DNA that enters a single polyplex at both room temperature (25 °C) and at near‐body temperature (37 °C), compare DNA encapsulation efficacy based on DNA chain length and conformation, and evaluate cellular uptake in primary human‐derived macrophages and test the cell viability of the polyplex systems. Demonstrating a thorough quantitative evaluation of encapsulation and cellular uptake will be crucial for developing effective carrier systems for gene delivery in the future.

## 2 Results and Discussion

### 2.1 Polyplex Formation and dSTORM Imaging

To explore pNIPAM/bPEI ‐ DNA polyplex formation, we first use a double‐stranded linear DNA fragment, 500 bp long, labeled with Alexa 647 fluorophores. We employ PCR labeling with Alexa 647, as outlined in the Methods section. We anticipate that approximately 10 ±1% of the nucleotides in the linear DNA will be fully labeled. Detailed information on the labeling protocol and labeling efficiency is provided in the Experimental Section, along with Tables S2 and S3 (Supporting Information). For the plasmid DNA, the labeling density varied from 1.4% to 3.3%. Since the DNA is double stranded, the number of nucleotides is two times the number of base pairs. Therefore, the average number of fluorophores per DNA molecule should scale with the number of base pairs time the labeling density. Not all flurophores will be active, but we can estimate the maximal number of active fluorophores, which is approximately 100 for 500 bp DNA, 700 for a 3527 bp DNA and between 100 and 240 for a 3527 bp pDNA. When the negatively charged DNA and positively charged microgel undergo complexation, the complex formation is an entropy‐driven process.^[^ ^34^ ^]^ Commonly the polymer amine (N) to DNA phosphate (P) ratio (N/P ratio) is a pivotal parameter used to design polyplexes.

In this study, we prepared polyplexes with N/P ratios of 0.6, 1.6, and 2.3. We first characterized our polyplexes by performing zeta potential measurements. For all N/P ratios examined, the zeta potential was negative (see Figure S3, Supporting Information). At N/P ratios below 1, the polyplex is expected to exhibit a negative zeta potential, while above an N/P ratio of 1, the overall net charge should be positive. However, it has been demonstrated that due to the branched architecture of bPEI, the polyplex can remain negative at N/P ratios up to 3.^[^ ^12^, ^35^ ^]^ This is also expected in our microgel system, as the redistribution of positive charges within the microgel may be sterically hindered.

**Figure 1:**
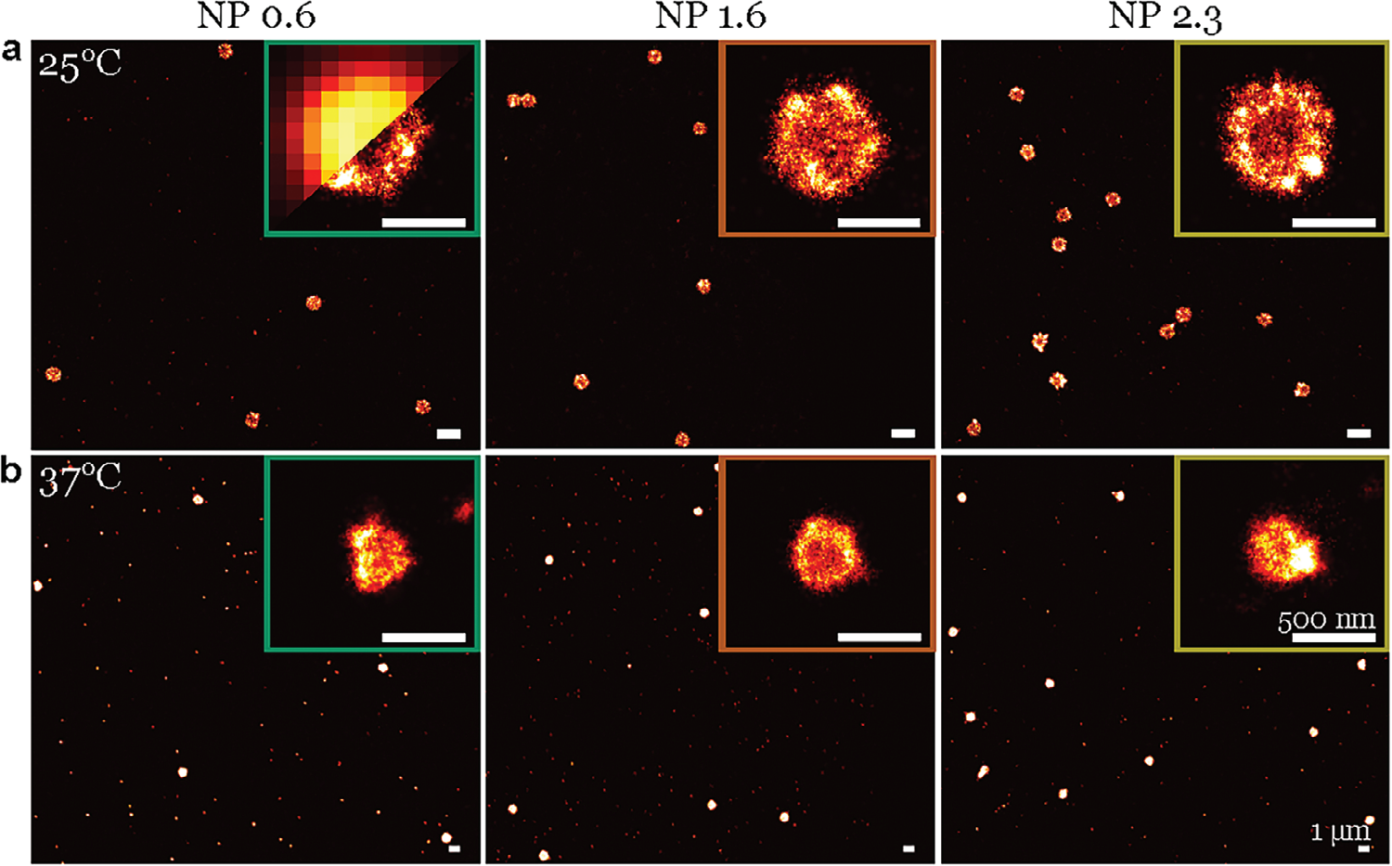
Super resolved images of Microgel‐DNA polyplexes. Polyplexes at N/P ratios 0.6, 1.6, 2.3 (left to right) a) at 25 °C and b) at 37 °C. The inset is an enlarged image of a single polyplex as resolved by super‐resolution microscopy. For comparison in the first inset, a diffraction‐limited image is included in the left upper corner of the same polyplex. In the Picasso software the parameter *“Maximum density”* was adjusted to 0.1 (a) and 0.25 (b).

We then employ dSTORM to investigate the differences between mixing ratios and the loading capacity of microgels at the single‐particle level. We investigate pNIPAM/bPEI‐DNA complexation under different conditions, i.e., mixing ratios and temperatures. First, to confirm the core‐shell structure of our microgels, we perform TEM imaging with inverse labeling (Figure S4, Supporting Information). Additionally, we conduct DLS measurements (Figure S2, Supporting Information) to confirm that the synthesized particles still exhibit a VPT around 33 °C and to determine the hydrodynamic radius of the particles. To perform the dSTORM experiments, we first immobilize the microgel polyplexes on a glass‐treated coverslip and immerse them in dSTORM imaging buffer injected into the sample chamber. In bright field images, the particles appear as dark spots (Figure S5a, Supporting Information). Switching to epifluorescence, the same spots become fluorescent (Figure S5b, Supporting Information), confirming that the microgels have been loaded with fluorescent‐labeled DNA. We then start dSTORM imaging by increasing the laser power to excite the dye molecules and induce controlled fluorescence intermittency, or “blinking,” of the dye.^[^ ^36^, ^37^ ^]^ We acquire 30 000 frames at 30 ms exposure time per measurement and proceed with dSTORM image analysis using the open‐source software *Picasso*.^[^ ^38^, ^39^ ^]^ With a temperature controller in the super‐resolution microscopy setup we observe the VPT of polyplexes in situ. For each N/P ratio, we perform dSTORM experiments at 25 °C and 37 °C on the same sample. **Figure** **1** showcases super‐resolved images of the polyplexes at N/P ratios 0.6, 1.6, and 2.3, both at 25 °C and 37 °C, where the core‐shell structure of the polyplexes is visible. As we increase the temperature, the polyplex shrinks to about half its initial size. This is in accordance with the DLS measurements for the same system, where the hydrodynamic radius changes from $R_{H}^{\text{DLS}}=383$ nm at 25 °C to $R_{H}^{\text{DLS}}=220$ nm at 37 °C. For the N/P ratios studied here, the polyplex's shape and size remain similar. The subsequent section will explore our main result, focusing on the analysis leading to the quantification of DNA encapsulation.

### 2.2 Measuring DNA Strand Encapsulation in Polyplexes

It is crucial to obtain information at the single‐particle level, determine the destination of genetic material within a complex system, and establish the loading capacity of a particular nano‐carrier. In addition to the standard characterization techniques, such as dynamic light scattering (DLS) and electrophoretic mobility (EM), which provide ensemble averaged information about the complexes, many different advanced microscopy techniques have been explored to obtain single‐particle information.^[^ ^40^, ^41^ ^]^ In the present work, we employ super‐resolution microscopy (SRM), which has revolutionized the study of small entities within biological systems. It offers unprecedented insight into their structural details by achieving optical resolutions on the order of tens of nanometers or better.^[^ ^42^ ^]^ To comprehend the formation and fate of complex structures in biological media, Albertazzi et al. investigated the PLGA‐pDNA polyplex system using a type of SRM called direct stochastic optical reconstruction microscopy (dSTORM). Their findings demonstrate the potential of this technique to achieve a better understanding of polyplex formation.^[^ ^43^, ^44^ ^]^

We carefully designed a calibration experiment to establish dSTORM as a technique capable of quantifying our system's properties without bias.^[^ ^45^ ^]^ To assess the number of blinking events for an individual DNA strand, we prepare a dilute sample containing only fluorescent‐labeled DNA, ensuring that each DNA strand is well separated from the others. Subsequently, we perform a dSTORM measurement using the same parameters as detailed in Section 2.1. In the stochastic blinking of the fluorophores, a single blinking event can persist for anywhere from one to several frames, influenced by factors such as the “ON” duration relative to camera settings, exposure time, and frame rate. Using a custom‐made code (Matlab, MathWorks Inc., USA), detailed in the Figure S6 (Supporting Information), we bin the localizations in consecutive frames from the same blinking event and tally the number of linked localizations for each DNA strand, applying pre‐defined imaging parameters. Specifics regarding sample preparation and image acquisition can be found in Section S6 (Supporting Information).

**Figure 2:**
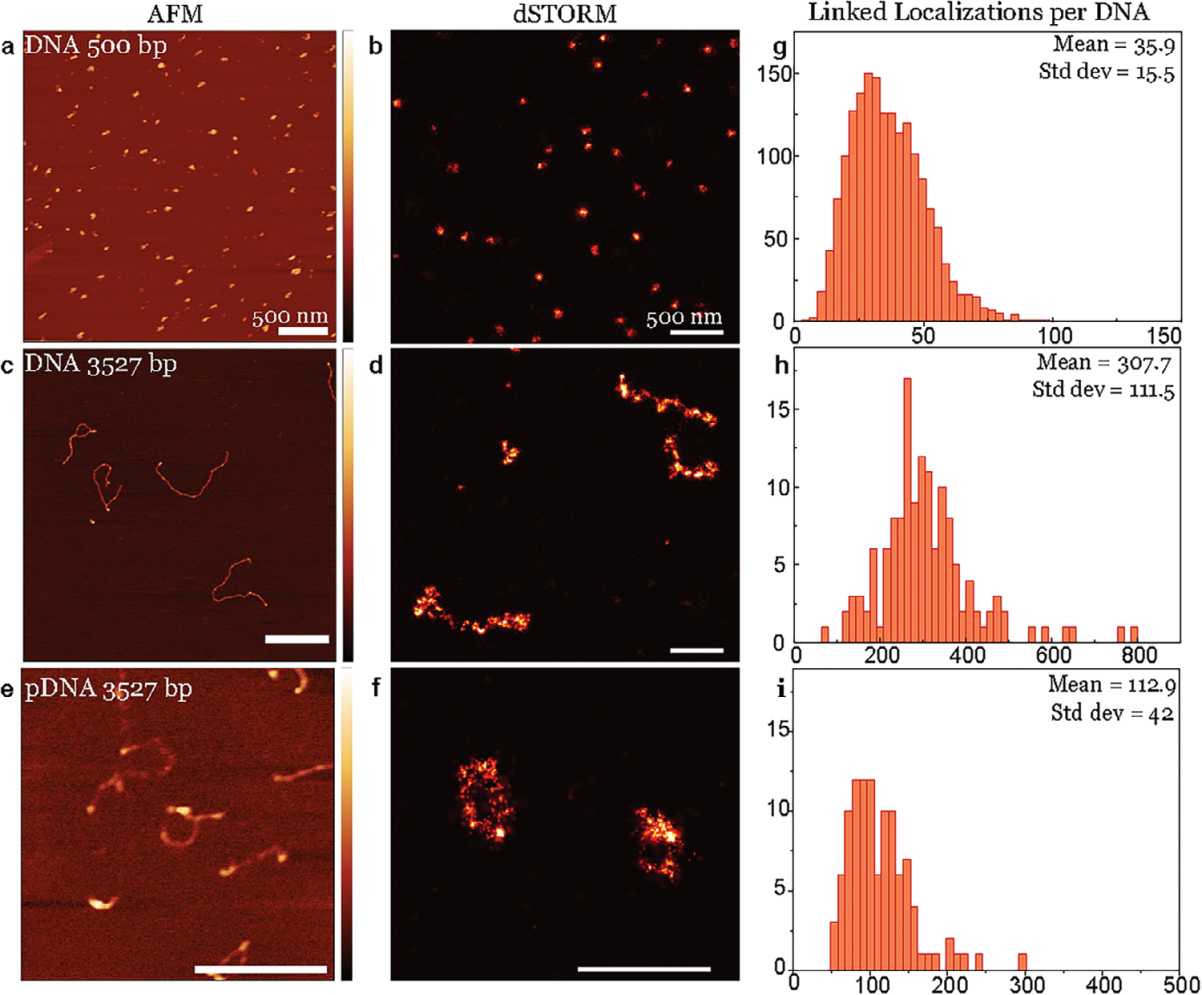
Calibration of imaging individual DNA strands. a–f) AFM (Colorbar 0–3 nm for a,c,e) and dSTORM images of individual DNA strands. g–i) Histograms of the number of linked localizations per DNA strand for DNA 500 bp, DNA 3527 bp, and pDNA 3527 bp. All scale bars represent 500 nm.

A crucial point in our image analysis is to be able to identify the signal of individual DNA strands and to exclude double and triple counts of the same DNA strands. To calibrate the dSTORM images, we conduct atomic force microscopy (AFM) measurements (**Figure** **2**a,c,e) for each type of DNA used in this study to determine the size and shape of individual DNA strands. We examine the three types of DNA introduced earlier: i) linear DNA 500 bp, ii) linear DNA 3527 bp, and iii) plasmid DNA 3527 bp. In Figure 2a,b (DNA 500 bp), we juxtapose the AFM results with dSTORM images captured under identical conditions and find that DNA 500 bp exhibits a size of approximately 100–150 nm in both the AFM and dSTORM images, displaying a similar configuration. The other two systems exhibit qualitatively similar shapes in both AFM and dSTORM. When elongated, a linear DNA strand of 3527 base pairs reaches a size of approximately 1000 nm (see Figure 2c,d). This measurement is consistent with the anticipated length, given that the well‐known spacing of DNA base pairs is 0.34 nanometers, which would result in an approximate length of 1200 nanometers for a 3527 base pair DNA strand. This reasoning also extends to the pDNA 3527 bp shown in Figure 2e,f, where the plasmid DNA adopts diverse loop configurations while retaining a comparable size in both AFM and dSTORM. The histograms in Figure 2g,h,i, illustrate the count of linked localizations for several hundred individual DNA strands analyzed.

With these measurements, knowing the number of detectable fluorophore localizations per DNA strand, we have established a calibration baseline that allows us to quantify our polyplexes. Ensuring consistent blinking kinetics of DNA, even when complexed with the microgel, we conducted a control measurement with one DNA per polyplex, following the approach by Albertazzi and coworkers.^[^ ^43^, ^44^ ^]^ We analyzed super‐resolved images of polyplexes, where most contained 0 DNA encapsulated, and only a few contained 1 DNA per polyplex (Figure S8, Supporting Information). Comparing consecutive frame lengths (number of frames one fluorophore stays on), we confirmed that the DNA blinking rate remained consistent both inside and outside of the microgel (Figure S9, Supporting Information), and the number of localizations per DNA was similar to the calibration measurement of DNA alone. In addition, we perform DNA calibration measurements at 37 °C to ensure that there are no temperature‐dependent changes in the measurement. The experimental results confirm this assumption (Figure S7, Supporting Information).

The precise calibration of dSTORM images allows us to conduct a quantitative analysis of DNA uptake in N/P 0.6, N/P 1.6, and N/P 2.3 polyplexes at different temperatures 25 °C and 37 °C. In a single polyplex at 25 °C, we can accommodate approximately 30–35 DNA 500 bp strands, corresponding to images shown in Figure 1a. As we increase the N/P ratio, there is no significant change in the number of encapsulated DNA strands. Moreover, for all N/P ratios, we observe residual uncomplexed DNA in the solution (see Figure S10, Supporting Information). While this is not the primary focus of the study, it highlights the potential of dSTORM to fine‐tune mixing ratios, achieving the optimal polyplex solution with maximum loading capacity and minimal uncomplexed DNA in the solution. When the temperature is increased to 37 °C, the polyplexes shrink; however, we find that the amount of encapsulated DNA remains constant. This demonstrates the system's capability to condense and protect the DNA at near body temperature.

Given that the encapsulation efficiency shows minimal variation in the number of DNA molecules per microgel across the different N/P ratios, we have chosen to proceed with an N/P ratio of 1.6 when exploring the different DNA lengths and shapes in the complex formation.

### 2.3 Encapsulation Efficiency When Varying DNA Chain Length and Shape

**Figure 3:**
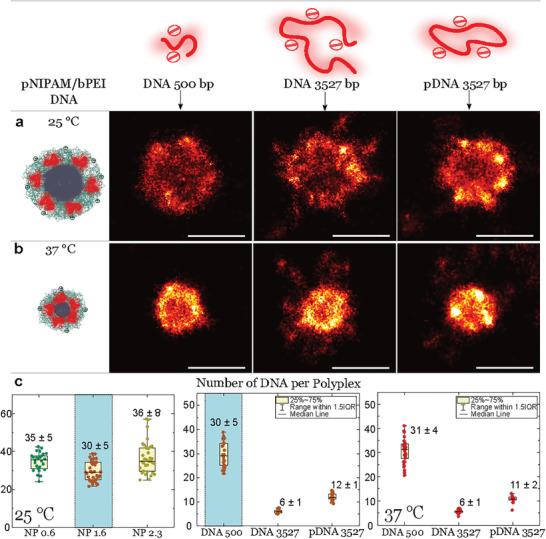
DNA encapsulation in pNIPAM/PEI carrier system. a) Polyplexes formed at 25 °C (from left to right) schematic illustration, dSTORM images of single polyplex formed with DNA 500 bp, DNA 3527 bp, and pDNA3527 bp. b) Polyplexes formed at 37 °C (from left to right) schematic illustration, dSTORM images of single polyplex formed with DNA 500 bp, DNA 3527 bp, and pDNA 3527 bp. Resolution set to individual localization precision iso (Picassso) for all dSTORM images and the maximum density is adjusted for all to 0.25. Scale bar 500 nm. c) The quantification graph shows in column bars the number of encapsulated DNA for different N/P ratio (500bp, 25 °C) and for the different DNA polyplex systems at constant N/P ratio 1.6 for 25 °C and 37 °C.

Studying the effectiveness of cell uptake of genetic material, especially when altering DNA length and shape, is crucial for enhancing gene carrier systems. In our research, we closely examine individual carrier particles to observe how their structure and DNA transport capacity vary with different DNA lengths and shapes. We used images and measurements of DNA taken with AFM and dSTORM (see Figure 2) to gain insights into the shapes and conformations of DNA molecules. We then prepare polyplexes with a ratio of N/P 1.6 for each DNA type and analyze them with dSTORM at 25° and 37°. We analyzed 20–30 particles at each temperature setting. In **Figure** **3**, panels a and b, we show super‐resolved images of the complexes for DNA with a length of 500 bp, 3527 bp, and pDNA 3527 bp.

These images help us understand how the structure of the polyplexes changes with varying DNA lengths and shapes, particularly under temperature variations. When measured at 25 °C, we find that the polyplexes with DNA 500 bp are smaller and more compact, while polyplexes with linear DNA 3527 bp show the presence of dangling ends of the DNA chains outside of the polyplex. Interestingly, when using the same DNA length in the form of plasmid DNA 3527 bp we observe more patches formed in the shell of the polyplex with almost no dangling ends sticking out of the complex. When the temperature is increased to 37 °C the pNIPAM/bPEI‐DNA system collapses, and the polyplexes with linear DNA 500 bp shrink to about ∼200–250 nm in radius. The polyplexes formed with linear DNA of 3527 bp, although shrinking to a similar size as DNA of 500 bp, exhibit dangling DNA chains extending several hundred nanometers outside the polyplex. In contrast, polyplexes formed with plasmid DNA of 3527 bp appear more compact, with fewer DNA chains protruding. It's important to note that these dangling ends can interact with the surface on which the polyplex is situated. Depending on the strength of interaction with the surface, the dangling chain may not collapse with the shrinking polyplex but remain attached to the surface.^[^ ^46^ ^]^

The graphs in Figure 3, panel (c), illustrate our quantitative estimation of the number of encapsulated DNA for various N/P ratios for linear DNA (500 bp) and for an N/P ratio of 1.6 across all DNA types and temperatures. For all three DNA systems, elevating the temperature to near body temperature only influences the size of the polyplex but not the DNA content. The DNA is more strongly condensed, but the number of encapsulated DNA molecules remains constant, indicating that there is no leak or release from the polyplex system. When comparing the number of encapsulated DNAs per polyplex, as anticipated, shorter linear DNA (500 bp) allows for the encapsulation of up to five times more DNA than longer linear DNA (3527 bp), while the total mass of complexed DNA remains nearly constant. Additionally, for the same DNA chain length, a comparison between linear and plasmid conformations reveals that under identical conditions, double the amount of plasmid DNA is encapsulated in these polyplexes. This is likely due to the circular conformation of the plasmid DNA, which can condense more effectively compared to its linear counterpart.^[^ ^24^, ^47^ ^]^

### 2.4 Interaction and Uptake of DNA Polyplexes by Primary Human‐Derived Macrophages

Demonstrating cellular uptake is crucial for establishing the potency of gene delivery systems and for understanding nanomaterials accumulation and trafficking pathways.^[^ ^48^, ^49^, ^50^ ^]^ It is well‐established that nanomaterial uptake and intracellular fate can vary based on their physicochemical properties, such as core material, size, shape, surface charge, porosity, surface roughness, surface area, and crystalline structure, which determine their interactions with surrounding proteins and the formation of a protein corona.^[^ ^51^, ^52^, ^53^, ^54^ ^]^ Here, we use confocal laser scanning microscopy (CLSM) to observe the uptake of the different polyplexes and monitor the photon count of these polyplexes within single cells. To ensure consistency and control for biological variability linked to cell division, we specifically selected non‐dividing cells.^[^ ^52^, ^55^, ^56^, ^57^ ^]^ We employed human monocyte‐derived macrophages exhibiting an M2‐like phenotype (MDMs), which are known for their anti‐inflammatory characteristics and exhibit robust phagocytic and endocytic capacities.^[^ ^58^ ^]^

**Figure 4:**
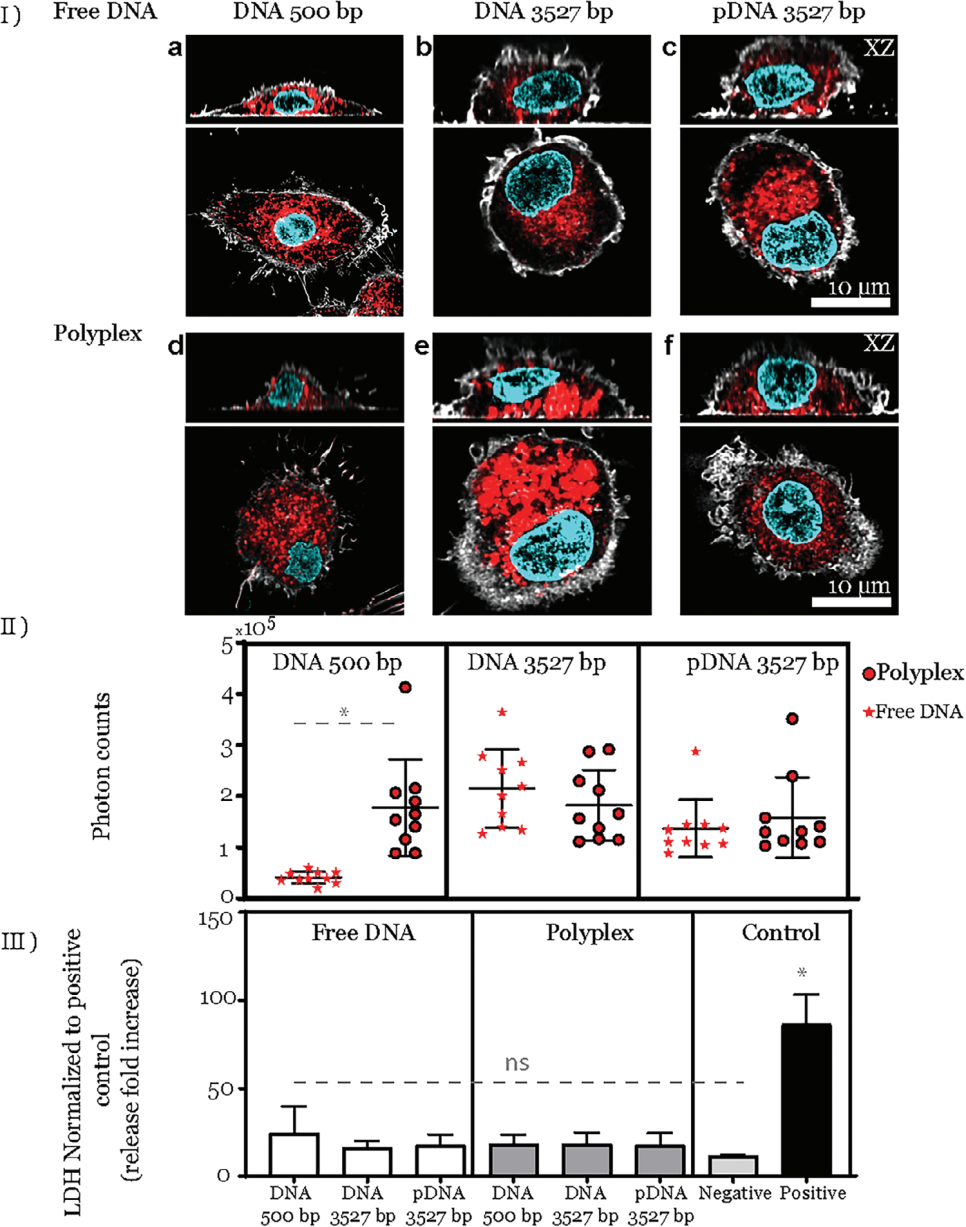
Uptake of DNA polyplexes by primary human‐derived macrophages. I) Representative CLSM and corresponding xz projections illustrate the uptake of free DNA (a–c) and polyplexes (d–f). The cell's cytoskeleton was stained to visualize F‐actin, shown in gray, and the nucleus was stained with DAPI, depicted in cyan. Polyplexes and free DNA within the cell are visualized in red. II) Photon counts per 10 individual cells analyzed for each condition: free DNA (stars) and DNA‐containing polyplexes (dots). Each data point represents an individual cell analyzed using FIJI. III) Cytotoxicity of DNA and polyplexes, determined by the LDH assay. Supernatants from non‐exposed cells served as controls. Data were normalized to the positive control and expressed as a fold increase in LDH release over the positive controls. Triton X‐100 (0.2%) served as a positive control for membrane rupture. Each condition had three biological replicates. All data correspond to MDMs exposed to 12 ng mL^−1^ of free DNA or polyplexes for 6 h, followed by a post‐exposure period of 36 h. The whiskers are the standard deviations. ^*^ *p* < 0.05 is the significant difference and (ns) non‐significant difference analyzed for photon counting with Welch's *t*‐test and for LDH by One‐way ANOVA.

Our experimental approach aimed to determine whether macrophages take up polyplexes over time and, if so, compare particle‐mediated delivery with the uptake of free DNA. To this end, we exposed MDMs to free DNA and DNA‐loaded polyplexes at concentrations of 12 ng mL^−1^ for 6 h, followed by 36 h post‐exposure. During this phase, we confirmed that both DNA and polyplexes were taken up by the cells, as shown in **Figure** **4**(I)a–f. We noticed a high accumulation of these samples after 6 hours, suggesting that polyplexes are taken up and are likely to be sorted and localized within the endolysosomal pathway.^[^ ^52^, ^55^, ^59^ ^]^ After 36 h post‐exposure, we observed no colocalization with the nuclei (Figure S11, Supporting Information). To compare the amount of DNA inside the cells, we set the confocal microscope to photon counting mode and acquired image stacks, confirming the uptake of DNA polyplexes by the MDMs. The polyplexes loaded with DNA 500 bp displayed an approximately fourfold increase in total photon counts per cell analyzed compared to the free DNA 500 bp. When comparing the photon counts of free DNA 3527 bp and pDNA with their respective polyplexes no significant change was observed. Despite the standard deviation in the photon counts per cell, stemming from cell‐to‐cell variability, the trend clearly shows increased uptake of DNA (500 bp) when using the polyplex delivery system. Moreover, the similar uptake rates for both free DNA and the other DNA polyplexes can be attributed to the similar interaction of the negatively charged polyplex and free DNA with the proteins in the cell culture medium, as well as the interaction with the cell membranes, further explaining the comparable uptake by macrophages, which are well known for their phagocytic and endocytic capacities.^[^ ^53^, ^54^, ^60^, ^61^ ^]^

Finally, we also verified that the concentrations did not induce cytotoxicity within the investigated time frame. We evaluated this using the lactate dehydrogenase (LDH) assay (Figure 4(III)), and we did not observe any significant changes in LDH release in the medium from cells exposed to DNA and polyplexes compared to control cells.

## 3 Conclusion

By employing advanced imaging techniques such as dSTORM, AFM, and CLSM, we were able to characterize individual DNA molecules and monitor the polyplex architecture with unprecedented clarity, on the molecular level. We were able to show that temperature and the N/P ratio do not substantially affect the loading capacity of polyplexes for the N/P ratios investigated here. Notably, we found that the number of DNA molecules encapsulated in a polyplex does not change with an increase in temperature. This suggests no leaks in the polyplex system and establishes them as stable carriers near body temperature. Moreover, our findings reveal that the choice of DNA length and shape significantly impacts the final structure and the loading capacity of polyplexes. We observed that shorter linear DNA molecules allowed for the encapsulation of a greater number of DNA strands compared to longer linear DNA molecules. Furthermore, dSTORM reveals the longer DNA chains protruding outside the complex, influencing the polyplex's final architecture. Interestingly, the plasmid DNA exhibited a higher encapsulation efficiency than linear DNA under identical conditions, highlighting the importance of considering DNA conformation in polyplex design. Our experimental study is consistent, and the evidence supporting our dSTORM approach for estimating the number of DNA molecules is convincing. For future work, orthogonal validation would be beneficial to independently corroborate our findings. This could include techniques such as dose‐response curves or quantitative PCR (qPCR) of the applied DNA‐polyplexes.

We demonstrated the successful cellular uptake of DNA polyplexes by MDMs without inducing cytotoxic effects. The uptake and retention in intracellular compartments of these polyplexes over time were confirmed, with significant accumulation observed within 6 h and persistence in intracellular compartments after 36 h post‐exposure. Moreover, analysis of photon counts per cell suggested enhanced counts and higher DNA content in DNA 500 bp polyplexes compared to free DNA 500 bp, indicating improved uptake efficiency, while there was no significant difference in the uptake efficiency when we compared DNA 3527 bp and pDNA3527 bp. In future studies, it would be advantageous to use smaller monodisperse microgel particles. This could enhance the likelihood of endosomal release, allowing the DNA to reach the nucleus more effectively.

Our findings demonstrate dSTORM super‐resolution microscopy as a quantitative tool for designing synthetic gene delivery carriers, which is a crucial contribution to advancing non‐viral gene delivery formulations for medical applications. We achieved these important results by elucidating, via dSTORM, the key factors influencing polyplex formation, such as N/P mixing ratios, DNA length, and conformation. These results highlight the importance of visualizing the complex formation at relevant conditions to develop efficient gene delivery systems.

## 4 Experimental Section

### Reagents

N,N‐Methylenebis(acrylamide) (BIS), branched poly(ethylenimine) (bPEI, MW 25 000 g mol^−1^), tert‐Butyl hydroperoxide (TBHP, 70% in water), Cysteamine (MEA), glucose oxidase from Aspergillus niger, catalase from bovine liver, glucose, HEPES and PBS were purchased from Sigma–Aldrich and used as received. N‐isopropylacrylamide was purchased from Acros Organics and recrystallized 3 times before use. The following plasmids were used: 4984 bp pGEX‐6P‐1 vector (cytiva 28954648) and 3527 bp pMax‐GFP (from Zachary Nagel & Leona Samson, Addgene plasmid 177825) expressing GFP under the control of the CMV promoter. For the cellular experiments, the following materials were used: Roswell Park Memorial Institute 1640 medium (RPMI; Cat.No. 11835030, Gibco, Life Technologies, Zug, Switzerland) supplemented with 10% v/v fetal bovine serum (FBS), 1% v/v penicillin/streptomycin, and 1% v/v L‐glutamine; Lymphoprep (Axis‐Shield, Oslo, Norway); CD14+ magnetic microbeads (Miltenyi Biotec, Germany); colony‐stimulating factor 1 (m‐CSF1, 10 ng mL^−1^, Miltenyi Biotec, Germany); human blood; Triton X‐100 (0.2% v/v in phosphate‐buffered saline (PBS), Merck, Switzerland); an LDH cytotoxicity detection kit (Roche Applied Science, Mannheim, Germany); Fluoromount Aqueous Mounting Medium (F4680‐25 mL, Sigma–Aldrich); 4% paraformaldehyde (in PBS, v/v); and immersion oil (Leica Microsystems, type N and F) with a refractive index of 1.52.

### PNIPAM/bPEI Synthesis

PNIPAM/bPEI microgels were synthesized by radical copolymerization in aqueous solution as previously described by Zhang et al.^[^ ^28^ ^]^ N‐isopropylacrylamide (NIPAM), is the monomeric unit which is re‐crystallized in hexane prior to use and N,N‐Methylenebis(acrylamide) (BIS) was used as the cross‐linker. In addition, branched poly(ethyleneimine) (bPEI, MW 25 000 g mol^−1^, diluted 50 wt% in aqueous solution), was added as a co‐monomer to be grafted around the pNIPAM core structure to incorporate free amine groups into the microgel network. tert‐Butyl hydroperoxide (TBHP, 70% in water) was used to initiate the polymerization. Typically, in a two‐neck round bottom flask, PEI solution (0.84 g) (pH adjusted with HCl 1M to 8) is mixed with NIPAM (0.48 g) and BIS (0.0066 g) in 25 mL Mili‐Q H_2_O. The reaction mixture was purged with nitrogen for 30 min before the temperature was raised to 70 °C. Next, 0.25 mL 0.01M TBHP was added to the reaction mixture and the temperature is increased to 80 °C. Five minutes after the initiator was added and the solution started to turn white. The reaction mixture was kept at 80 °C for 4 h before rapidly cooling down in an ice bath. Following this protocol PNIPAM/bPEI core shell microgels are formed with PEI only being present on the shell of the microgel network. A purification step follows to remove all unreacted monomers in the solution by centrifugation and dialysis.

### DNA Labeling

For PCR labeling of linear DNA, the “HighFidelity AF647 PCR Labeling Kit” (Jena Bioscience) was used. The 500 bp DNA fragment was amplified from the Lambda DNA template provided as control with the corresponding primers from the kit. The primer pairs listed in Table S1 (Supporting Information) were utilized to amplify the other fragments. The reaction conditions were used according to the “Standard PCR labeling protocol” from the kit with a threefold increase of all reagents resulting in a total volume of 60 µL. Cycling conditions were used as proposed by the standard protocol but the cycle number was increased to 35. The labeled PCR products were purified with the QIAquick®PCR Purification Kit (QIAGEN 28104) and eluted from the columns with 2x30µL of EB buffer. Both eluates were pooled and the concentration determined with a nanodrop spectrophotometer.For labeling of plasmid DNA, the “Label IT®TrackerTM Intracellular Nucleic Acid Localization Kit, Cy®5” (Mirus MIR 7021) was used to label 5 µg of pMax‐GFP plasmid DNA with Label IT®TrackerTM reagent at a 1:1 (v:w) ratio. The reaction was incubated for 2.5 h at 37 °C. The resulting plasmid was purified by ethanol precipitation and resuspended in 50 µl of water.

### Polyplex Formation

Polyplexes were formed by mixing PNIPAM/bPEI microgel stock solution with bPEI concentration (2.5 ug mL^−1^) and stock solutions of DNA with lengths of 500 bp (30 ng uL^−1^), 3527 bp (72 ng uL^−1^), and plasmid DNA 3527 bp (115 ng uL^−1^) with varying concentrations to reach a final DNA concentration of 3 ug mL^−1^ in a total volume of 0.1 mL for each polyplex solution in aqueous media (MilliQ water Q‐POD®). To alter the N/P ratio the DNA concentration was kept constant while the microgel concentration was increased accordingly. The following steps were followed rigorously. First, the aqueous microgel dilutions were prepared simultaneously for three N/P ratios (0.6, 1.6, 2.3), each with a final volume of 50 uL. The solution was vortexed gently for 30 s. Next, the aqueous DNA dilution was prepared to a final volume of 50 uL. The solution was vortexed gently for 30 s. The DNA solution was added to the microgel solution by simple dipping for a total polyplex volume of 100 uL and DNA concentration of 3 ug mL^−1^. Additionally, 1 uL of HEPES buffer pH 7.4 (1 M) was added. The polyplex solution was placed in an oscillating tray (with a slow movement of 42 oscillations per minute) for 4 h at room temperature before the measurement in dSTORM. The sample was refrigerated, and additional measurements were conducted after 24 and 48 h using dSTORM. Detailed calculations of the N/P ratio and the estimation of bPEI concentration are provided in Section S1 (Supporting Information).

### Polyplex Sample Preparation and Image Acquisition

Coverslips (22 mm × 22 mm, #1.5) were sonicated in isopropanol for 10 min and blow air dried. Microscopy slides (76 mm × 76 mm, thickness 1mm) were cleaned using ethanol‐soaked tissue. The cover‐slips were treated using Ozone (PSD pro series, Novascan) for 5 min. An imaging chamber was created by attaching a microscopy slide to the coverslip using a strong double‐sided adhesive. The chamber volume was determined to be approximately 20 µL. Approximately 20 µL of the polyplex was incubated in the chamber for 20–25 min. This was followed by rinsing with 80 µL of dSTORM buffer (50 mM Tris, 10 mM NaCl, 10% w/v glucose, 50 mM cysteamine, 0.5 mg/mL glucose oxidase, 40µg mL^−1^ glucose catalase) and it was sealed using Tweensil glue.

To perform dSTORM experiments, a Nikon Ti‐Eclipse inverted microscope with an EMCCD camera (Andor iXon Ultra 897) Ti‐ TIRF illuminator unit was used to measure in highly inclined and laminated optical sheet (HILO) to achieve highly inclined illumination and limit the fluorescence background noise. A continuous wave red laser, coherent Genesis MX‐STM with 1 W output power at 639 nm was used, providing a single mode TEM00 Gaussian beam, horizontally polarized. The high‐power red laser enables fluorophores in their excited state, through inter‐system crossing, to occupy the triplet state where they get trapped. The laser was coupled into a single‐mode fiber (S405XP, Thorlabs) into the TIRF arm. The light was focused on the back aperture of a high numerical aperture and magnification objective (NA 1.49 and 100x magnification). With an extra zoom lens placed before the camera, the final pixel size was determined to be 107 nm. A dichroic filter with a wavelength 700 nm, and bandwidth of 50 nm is placed in the detection pathway (ET700/50, Chroma). We employ a high laser power(∼2 kW cm^−2^) to induce stochastic blinking to bring the fluorophores to a metastable dark state. dSTORM images were acquired for 30 000 frames at 30 ms exposure time. For these experiments AF647 was used, which is particularly noted for its high photostability and low tendency to self‐quench, even at high local concentrations, making it an ideal choice for super‐resolution microscopy. Previous work showed that comparable or higher density of AF647 led to reliable data with no detectable influence of, e.g., self‐quenching.^[^ ^62^ ^]^

### AFM Imaging

AFM imaging was carried out using the Bruker Dimension Icon AFM in PeakForce Tapping®mode under ambient conditions. A ScanAsyst‐Air cantilever (nominal spring constant of 0.4 N m^−1^) was employed for the measurements, with a setpoint force of approximately 0.5 nN and a peak force amplitude of 50 nm. The scanning rate was set at 1 Hz and a Peak Force Frequency of 1kHz was used during the measurements. The image analysis was done using the Gwyddion software.

### Cell Culture

Monocyte‐derived macrophages (MDMs) were obtained by isolating and differentiating human peripheral blood monocytes from human buffy coats (Interregionale Blutspende (donation service) SRK AG, Bern, Switzerland), following the protocol established by the Bionanomaterials research group at the Adolphe Merkle Institute. Human blood was initially processed using density gradient centrifugation with Lymphoprep (Grogg Chemie). The monocyte fraction was purified using CD14+ magnetic microbeads (Miltenyi Biotec, Germany). Differentiation was induced with colony‐stimulating factor 1 (m‐CSF1, 10 ng mL^−1^, Miltenyi Biotec, Germany) in Roswell Park Memorial Institute 1640 medium (RPMI; Cat.No. 11835030, Gibco, Life Technologies, Zug, Switzerland) supplemented with 10% v/v fetal bovine serum (FBS), 1% v/v penicillin/streptomycin, and 1% v/v L‐glutamine (cRPMI). The cells were cultured in 6‐well plates for 7 days for differentiation. Before seeding, the cell concentration in suspension was determined using an automated cell counter (EVE, NanoEnTek Inc., South Korea). A total of 100 000 cells/300 µL were used for each experiment. This was approved by the Federal Office for Public Health Switzerland committee (reference number: 611‐1, Meldung A110635/2).

### Cell Exposures and Uptake of Free DNA and DNA Polyplexes

A 3 ug mL^−1^ solution of free DNA and polyplexes (DNA 500, DNA 3500, pDNA 3500) was prepared in Milli‐Q water. This was diluted in cRPMI to a final concentration of 12 ng mL^−1^ and administered to the cells using a pre‐mixed method immediately before exposure for 6 h, followed by 36 h post‐incubation. Following sample exposure, the cells were washed with PBS and fixed with 4% paraformaldehyde (in PBS, v/v) for 1 h. After additional washes, cells were stained for F‐actin with a 0.66 µM Rhodamine‐Phalloidin probe (Cat. #R415, Invitrogen, Thermo Fisher Scientific Inc., Zug, Switzerland) for 1 h, and cell nuclei were stained with DAPI (1 µg mL^−1^ in PBS, Cat. #D9542, Sigma‐Aldrich, Buchs, Switzerland) for 10 min. After staining, cells were washed and mounted with Fluoromount (F4680, Sigma–Aldrich).

### Confocal Imaging Acquisition

Representative images were captured using a Leica Stellaris 5 confocal microscope (Leica Microsystems) equipped with HyD detectors. The microscope was set with an HC PL APO CS2 63x/1.40 OIL objective, and excitation lasers at 405 nm (DAPI), 561 nm (Rhodamine‐Phalloidin), and 638 nm (DNA and Polyplexes). Z‐stacks were collected and further processed using Fiji's ImageJ‐based software^[^ ^63^ ^]^ (National Institutes of Health, Bethesda, MD, USA). Photon counting imaging was performed with the following parameters: 16‐bit image (1024 × 1024 pixels), 2 Airy units, and HyD S 3 (643–750 nm) set to active photon counting mode. The gain was set at 2%, with the laser line (638 nm) intensity at 5%. All images were collected at 400 Hz, with 8‐frame accumulations per image and z‐stacks totaling 40 frames. Images were analyzed using Fiji with a custom‐written script. Each z‐stack was initially processed using a sum slices projection mode. Cells were manually defined using the Alexa 647 channel to establish the region of interest (ROI) for individual cell analysis. The photons detected in the Alexa 647 channel were masked, followed by automatic threshold adjustment. An inverted mask was applied to remove background noise and measure the raw integrated density, with ten individual cells analyzed for each sample exposure. The statistical analyses were conducted using GraphPad Prism 9 software and Origin 2016, 64‐bit, with each treatment adjusted to the appropriate statistical method. Analyses included Welch's *t*‐test. The results are presented as the mean with the standard deviation of the mean.

### Cell Viability

The supernatants' enzyme lactate dehydrogenase (LDH) levels were measured in triplicates for three independent replicates following the manufacturer's protocols (Roche Applied Science, Mannheim, Germany). LDH activity was measured to assess cytotoxicity in MDMs. The positive control for membrane rupture was 0.2 % Triton X‐100, diluted in the cell culture medium (v/v) for 24 h. The absorbance of the colorimetric product, formazan, was determined spectrophotometrically using a BioTek Synergy H1 spectrophotometer (Agilent Technologies, Basel, Switzerland) at 490 nm, with a reference wavelength of 630 nm. LDH values are presented as a fold increase relative to the positive control.The statistical analyses were conducted using GraphPad Prism 9 software and Origin 2016, 64‐bit, with each treatment adjusted to the appropriate statistical method. Analyses included one‐way ANOVA. The results are presented as the mean with the standard deviation of the mean.

## Conflict of Interest

The authors declare no conflict of interest.

## Author Contributions

X.S. conceived the study and and performed most of the dSTORM experiments and data analysis under the supervision of F.S. E.W., and M.A. contributed to the dSTORM experiments. C.F. handled all DNA labeling and PCR. S.N.R. conducted the AFM measurements. A.M.M. performed the cellular uptake experiments using the confocal microscope under B.R.R.'s supervision. A.M.M., D.V.H., and B.R.R. participated in confocal microscopy data analysis and interpretation. X.S., A.M.M., and F.S. wrote the manuscript with contributions from all authors.

## Supporting information

Supporting Information

## Data Availability

The data that support the findings of this study are available from the corresponding author upon reasonable request.
