# Supplementary material for: Polymer Nano‐Carrier‐Mediated Gene Delivery: Visualizing and Quantifying DNA Encapsulation Using dSTORM
Source: Small. 2024 Nov 17;21(1):2405929. doi: 10.1002/smll.202405929 (PMC11707562; doi:10.1002/smll.202405929)
Supplement: Supplementary file 1 — Supporting Information [file SMLL-21-2405929-s001.pdf]

NANO · MICRO
small

Supporting Information

for *Small*, DOI 10.1002/smll.202405929

Polymer Nano-Carrier-Mediated Gene Delivery: Visualizing and Quantifying DNA Encapsulation Using dSTORM

*Xhorxhina Shaulli, Aura Maria Moreno-Echeverri, Mariza Andoni, Eileen Waeber, Shivaprakash N. Ramakrishna, Cornelia Fritsch, Dimitri Vanhecke, Barbara Rothen-Rutishauser and Frank Scheffold**

Supplementary Information for:
Polymer Nano-Carrier-Mediated Gene Delivery: Visualizing and Quantifying DNA Encapsulation using dSTORM

Xhorxhina Shaulli,[1] Aura Maria Moreno Echeverri,[2] Mariza Andoni,[1] Eileen Waeber,[1] Shivaprakash N. Ramakrishna,[3] Cornelia Fritsch,[4] Dimitri Vanhecke,[2] Barbara Rothen-Rutishauser,[2] and Frank Scheffold[1, a)]

[1)] *Department of Physics, University of Fribourg, Chemin du Musée 3, 1700, Fribourg, Switzerland*

[2)] *Adolphe Merkle Institute, Chemin des Verdiers 4, 1700 Fribourg, Switzerland*

[3)] *Department of Materials, ETH Zürich, Vladimir-Prelog-Weg 1-5/10, 8093 Zürich, Switzerland*

[4)] *Department of Biology, University of Fribourg, Chemin du Musée 10, 1700 Fribourg, Switzerland*

[a)] Electronic mail: frank.scheffold@unifr.ch

LIST OF FIGURES

S1 1H NMR Analysis . . . 3
S2 DLS measurements . . . 4
S3 Zeta potential measurements . . . 4
S4 TEM imaging . . . 5
S5 From brightfield to super-resolved images of Polyplexes - Workflow . . . 6
S6 Linked localizations per DNA molecule- Workflow . . . 8
S7 25 vs 37 . . . 9
S8 1 DNA per polyplex . . . 10
S9 Consistency in blinking behavior . . . 11
S10 Non-complexed DNA in the polyplex solution . . . 11
S11 Cellular uptake and colocalization . . . 12

I. N/P RATIO CALCULATION

For all formed polyplexes the number of amine groups in the polymer to the number of phosphate groups in the nucleic acid, the N/P ratio is calculated as:

$$N/P = \frac{C(N)}{C(P)} \tag{S1}$$

The number of charged phosphate groups of DNA present in solution is estimated by:

$$C(P) = \frac{C_{DNA}}{MW_{bp}} \times 2 \tag{S2}$$

where MW_{bp}=660 g $\cdot$ mol^{-1} is the average bp molecular weight and C_{DNA} is the concentration of DNA in solution in g$\cdot$L^{-1}. The factor 2 is a result of the two phosphates per bp of DNA. The concentration of DNA is calculated using a NanoDrop spectrophotometer.

The number of charged amino groups for bPEI present in solution is estimated by:

$$C(N) = \frac{C_{bPEI}}{MW_{bPEI}} \times 1 \tag{S3}$$

where MW_{bPEI}=43 g $\cdot$ mol^{-1} and C_{bPEI} is the concentration of bPEI in solution in g$\cdot$L^{-1}. The factor 1 appears as there is approximately one charged amine per building block. The concentration of bPEI is difficult to calculate since there are unreacted monomers at the end of the synthesis which are then removed by centrifugation and dialysis. Initially, the concentration of the purified solution was determined to be approximately 11 mg$\cdot$ mL $^{-1}$ by lyophilization.

Based on the total concentration of the microgel, we can deduce the proportion of bPEI present. To achieve this, we conducted ^{1}H NMR measurements, which allowed us to calculate the concentration of bPEI as follows:

$$\text{bPEI\%} = \left(\frac{\frac{\int_s PEI}{H_s^+ PEI}}{\frac{\int_s PNIPAM}{H_s^+ PNIPAM} + \frac{\int_s PEI}{H_s^+ PEI}} \right) \times 100$$

$$\text{bPEI\%} = \left(\frac{\frac{0.61}{2H^+}}{\frac{1.01}{1H^+} + \frac{0.61}{2H^+}} \right) \times 100$$

$$\text{bPEI\%} = \left(\frac{\frac{0.61}{2}}{\frac{1.01}{1} + \frac{0.61}{2}} \right) \times 100$$

$$\text{bPEI\%} = \frac{0.305}{1.315} \times 100 = 23.19\%$$

This means that 23% of the total concentration (11 mg· mL $^{-1}$) comes from bPEI, meaning the C_{bPEI} is 2.5 mg· mL $^{-1}$. With this information, we can now calculate the N/P ratios.

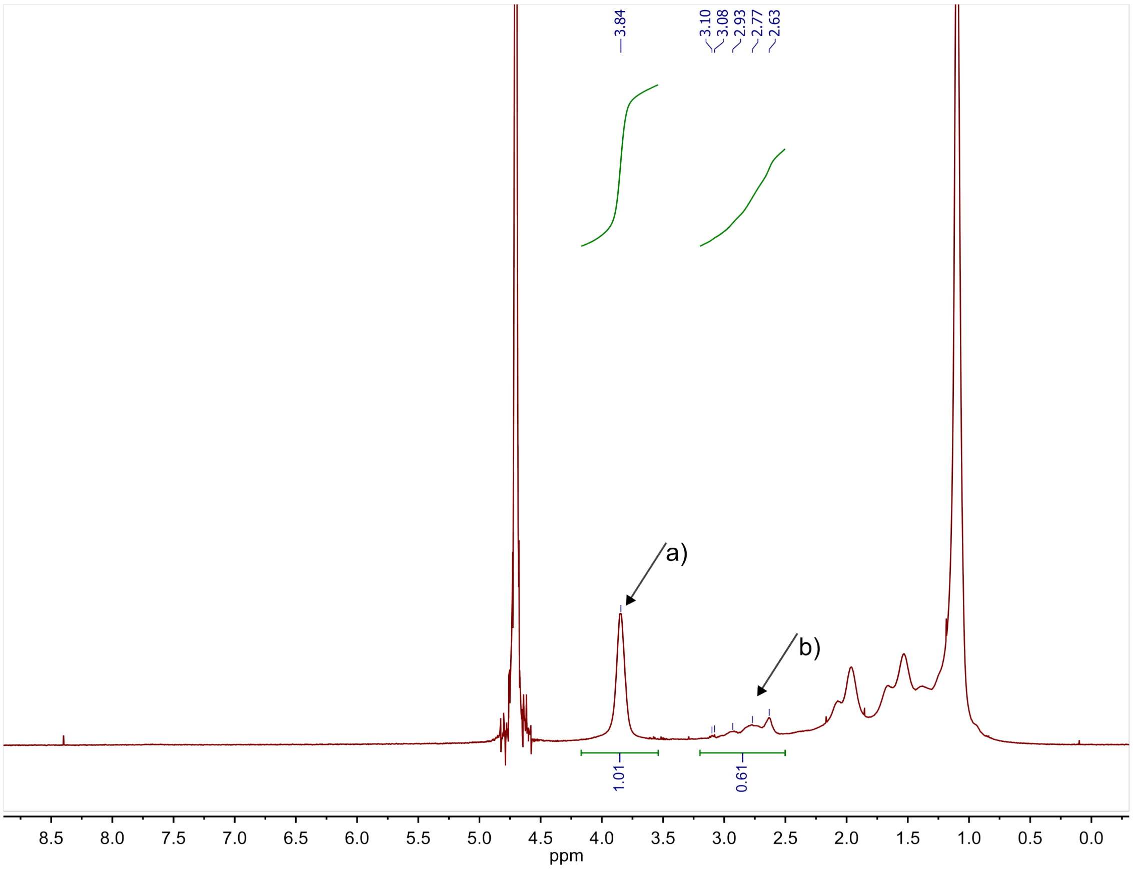

Figure S1: **1H NMR Analysis** 1H NMR spectra of PNIPAM/bPEI (D2O, 400 MHz).The average composition was determined from the integrals at a) 4.8 ppm corresponding to the signal of the proton from the -CH- of the isopropyl group of the PNIPAM. b) Peaks from 2.6 ppm to 3 ppm assigned to the -CH2-NH2 of the bPEI.

II. DLS

DLS measurements were performed using the commercial LS Spectrometer, 2D-DLS Pseudo cross-correlation set-up (LS Instruments AG, Switzerland). We increase the temperature from 25 to 39 °C with ΔT= 2 °C as step size. Laser light with wavelength of 660 nm was used to perform the experiments at scattering angles of 30 - 90° with Δ 5° as step size. Five measurements of 60 seconds were obtained at every angle. The diffusion coefficients D and hydrodynamic radii R_H^{DLS} were extracted from a multi angle analysis of the first order cumulant fit.

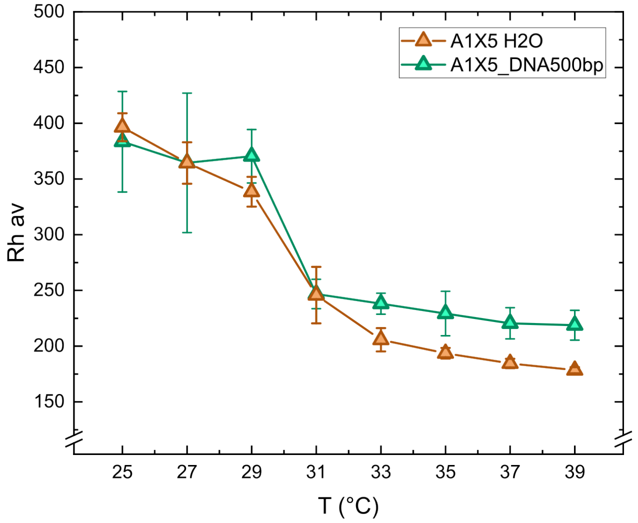

Figure S2: **DLS measurements** Hydrodynamic radius (nm) of pNIPAM/bPEI microgels (orange) and pNIPAM/bPEI-DNA 500 bp polyplexes(green) at temperatures 25 to 39 °C.

III. ZETA POTENTIAL MEASUREMENTS

Before complexation, PNIPAM/bPEI has a positive zeta potential (30 ± 1 mV). After complexation we performed zeta potential measurements for polyplexes with DNA 500 bp and pDNA 3527 bp for all N/P ratios studied here and the results are shown in figure S3.

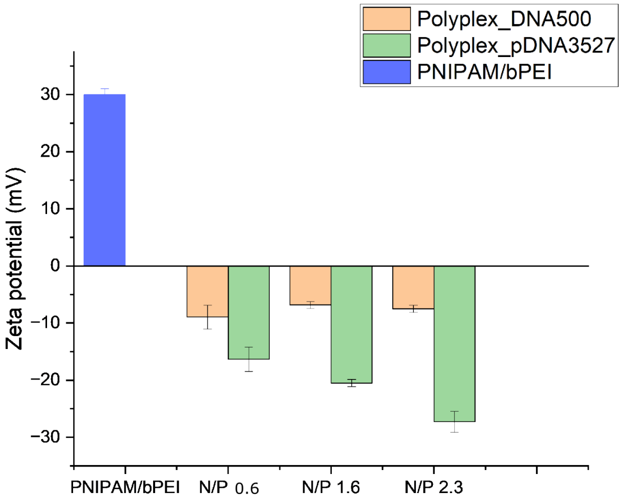

Figure S3: **Zeta potential measurements** Zeta potential measurements of the microgel PNIPAM/bPEI before polyplex formation and of all N/P ratios for polyplexes formed with linear DNA 500 bp and plasmid pDNA 3527 bp.

IV. TEM IMAGING

The core–shell morphology of the pNIPAM/bPEI was characterized using transmission electron microscopy (TEM; FEI Tecnai G2 Spirit, Thermo Fisher Scientific, Waltham, MA, USA). To prepare the samples, 5 μL of the microgel solution was deposited onto a 200 mesh

Formvar carbon-coated grid and allowed to settle for 5 minutes. The excess solution was then blotted away with filter paper. This was followed by the contrast stain which was done by the addition of 5 μL of UranyLess EM Stain (EMS, Hatfield, PA 19440) for contrast enhancement, left for 5 minutes before blotting off the excess stain with filter paper. The grid was then left to dry for 24 hours prior to microscopy observation.

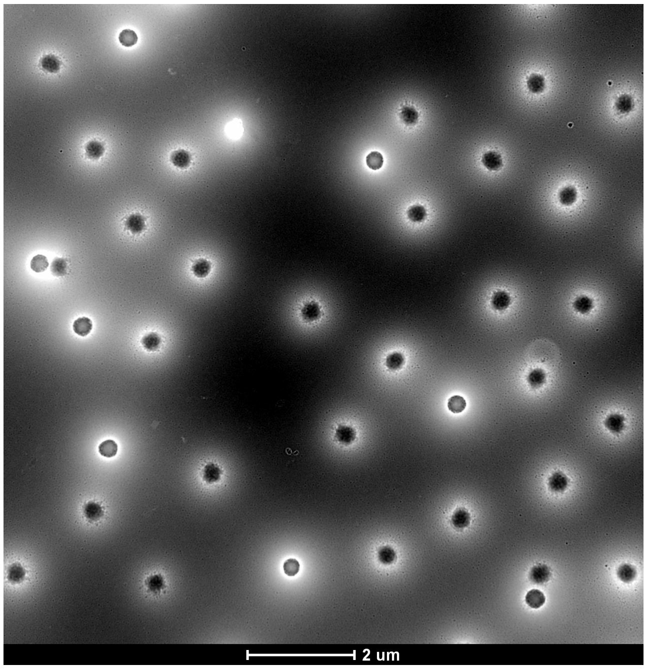

Figure S4: **TEM imaging** TEM image of core-shell pNIPAM/bPEI microgels where the pNIPAM core is seen in black while the bPEI shell in white as a result of the inverse labeling.

V. DIRECT STORM DATA ANALYSIS

First, dSTORM images were analyzed using the Picasso software, which is composed of several components that allow for the localization of individual blinking events in each frame. The initial step involves utilizing the "Localise" component, where we input specific camera parameters, such as EM gain (52), Baseline (55), Sensitivity (5), Quantum efficiency (0.93), Pixel size (107), and Magnification factor adapted following Bratton et al. 2015[1] (0.73). The true EM gain was extracted following Mortensen et al.[2] For fitting, we opted for the Maximum Likelihood Estimation (MLE) integration Gaussian method. After configuring these parameters, we preview the localized polyplexes and fine-tune the minimum net gradient to selectively identify strong blinking events. The chosen minimum net gradient typically ranges between 20,000 and 30,000, ensuring a robust selection of blinking events, as we intend to filter out unwanted events in subsequent steps. The second phase involves employing the "Filter" component, where we eliminate selected spots with either low or excessively high photon counts. Typically, we retain particles within the range of 500 to 30,000 counts. We also filter out elliptical particles with an ellipticity range larger than 0.6. The frames considered for this step span from 5000 to the final frame, and we apply a constraint on localization precision, setting it up to 0.4 pixels in both x and y dimensions. Typically the ellipticity and the localization precision can be set much lower as the data quality allows to do so, but we on purpose are more generous and lower the threshold to also account for the blinking events that might be slightly out of focus because of their z

position but still important to be accounted for. The concluding step is the rendering phase, where we visualize and post-process the gathered localizations. Drift correction is performed by choosing the "undrift by RCC" option with a segmentation of 1000. The hot colormap filter is applied, along with individual localization precision settings (iso). To analyze each individual polyplex, we utilize the pick tool with a diameter of 9 camera pixels. Upon selection, each polyplex is assigned a random color for visual representation. Secondly, we analyze the individual polyplexes by using an in-house MATLAB routine that performs the post-processing of the data as detailed in Figure S6

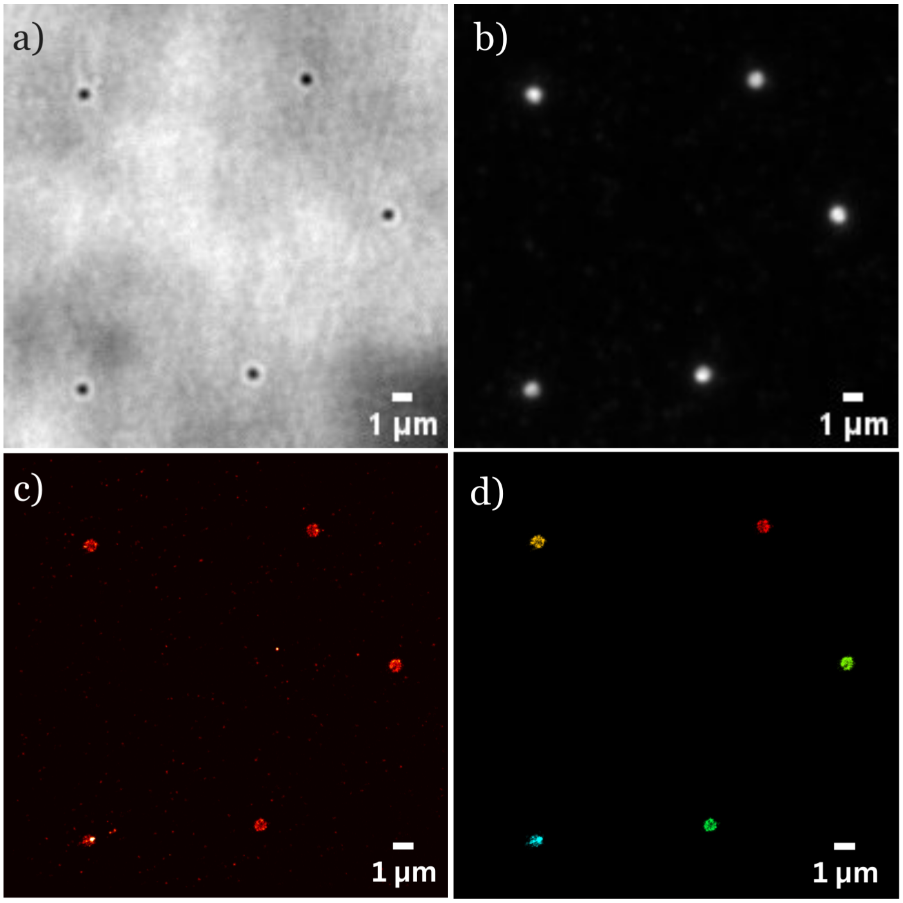

Figure S5: **From brightfield to super-resolved images of Polyplexes - Workflow** Workflow from brightfield to super-resolved images a) Brightfield microscopy image where we can easily distinguish the microgels, b) Epifluorescence microscopy image revealing the fluorescent DNA inside the microgels in the same locations as brightfield confirming polyplex formation c) dSTORM image of the polyplexes from Picasso software following polyplex localization, filtering, Rendering d) Render image of selected polyplexes with the pick tool, each uniquely color-coded for enhanced visualization.

VI. DNA ONLY MEASUREMENTS

The first control measurement we do to quantify the amount of DNA that goes inside a polyplex, is to calculate the mean number of linked localizations we get for a single DNA strand. To do this, measurements on individual DNA strands were performed for the respective DNAs used in this study (DNA 500 bp DNA3527 bp, and pDNA3527). The experimental

conditions were kept the same as for the polyplex experiments for temperatures of 25 and 37°C as explained in methods section of the main text. The measurements are followed by a general data analysis in Picasso as explained in the section V. For further analysis, we are interested in the number of localizations per DNA molecule. The treatment of the localizations obtained from the general analysis is illustrated in S6. Further explanation in this section refers to this visual. After the treatment of the super-resolution image, suitable DNA molecules are **(a)** selected according to certain criteria to have a certain size and shape based on the AFM images that we take for the same structures. The picked molecules are saved in a new file **(b)** where each group, i.e. DNA molecule, is assigned to a different color **(c)** in the dSTORM image. In the data file, a new row is added which allocates the localizations to their respective group. Using Matlab **(e)**, single and double frames are sorted out. Single frames possess no localization in the frame before and after therefore their short blinking time could introduce artifacts and double frames refer to frames that contain two or more localizations at the same time which can also interfere in finding with high precision the center of the blinking event. Each fluorophore emits photons over multiple consecutive frames that are, in the same step, joined together to linked localizations. One linked localization can therefore possess multiple frames. Groups that were first described with their total number of localizations **(d)** are now characterized using linked localizations **(f)**. The advantage of this step is the independence of the average consecutive frame length and number of localizations. Daily changes in the set-up or small variations in the buffer environment can influence the duration of a blinking event. This will have an impact on the average consecutive frame length per group and the total number of localizations per group which will make comparison to other measurements impossible. However, if the acquisition time is held constant and the blinking behavior of the dyes is not changed drastically, the total number of linked localizations per group should be constant. This makes linked localization a very useful quantity. This data analysis is followed for both DNA-only measurements and polyplexes and the number of linked localisation per DNA strand is used then to calculate the number of DNA molecules inside the polyplex.

A. Sample preparation

We diluted the DNA-only stock solution (concentration of 34.1 ng/uL for 500 bp and 25 ng/uL for 3527 bp) in MilliQ water to obtain a reasonable density of individual DNA strands in the FOV. To perform a successful dedrift during the image analysis, TetraSpeck beads (0.1uL) were added to the solution. Before the incubation of the final DNA solution, the imaging chamber was incubated with a Poly-L-Ornithine solution to achieve a positively charged coverslip surface. This will result in the DNA strands binding to the surface. Finally, the imaging chamber was washed with the dSTORM buffer prepared on that day to obtain the desired stochastic blinking of the dyes. The analysis process is identical to the procedure described in V. In the render module, however, the undrift is conducted by selecting TetraSpeck beads in the FOV as drift markers.

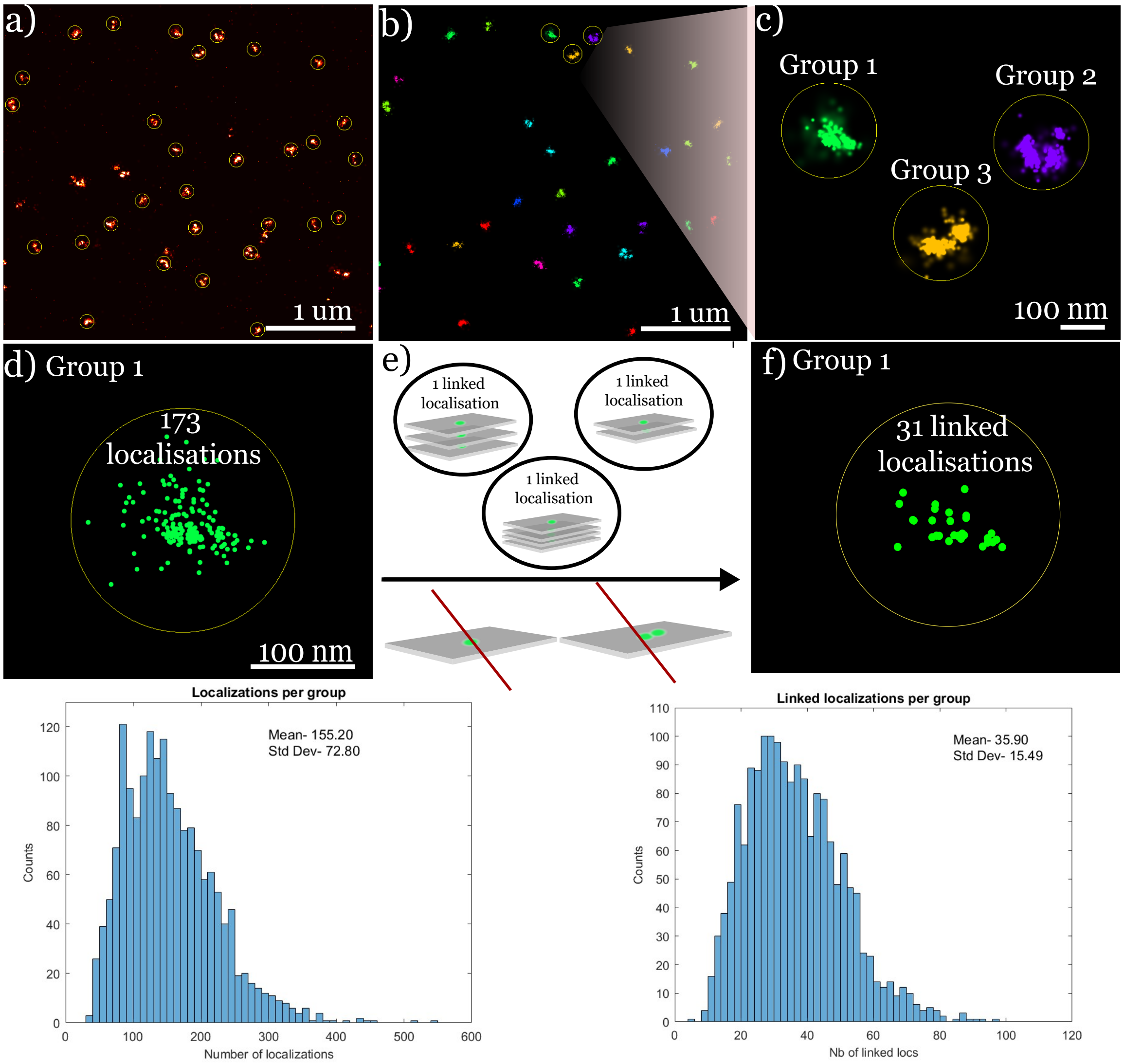

Figure S6: **Quantification for the number of localizations per DNA molecule** Number of localizations per DNA molecule was estimated after careful analysis, starting with **(a)** picking suitable DNA strands to sort them into groups (depicted on **(b)** and **(c)**). Localizations of each group are filtered to sort out single and double frames, followed by grouping consecutive frames to linked localizations as shown in **(e)**.

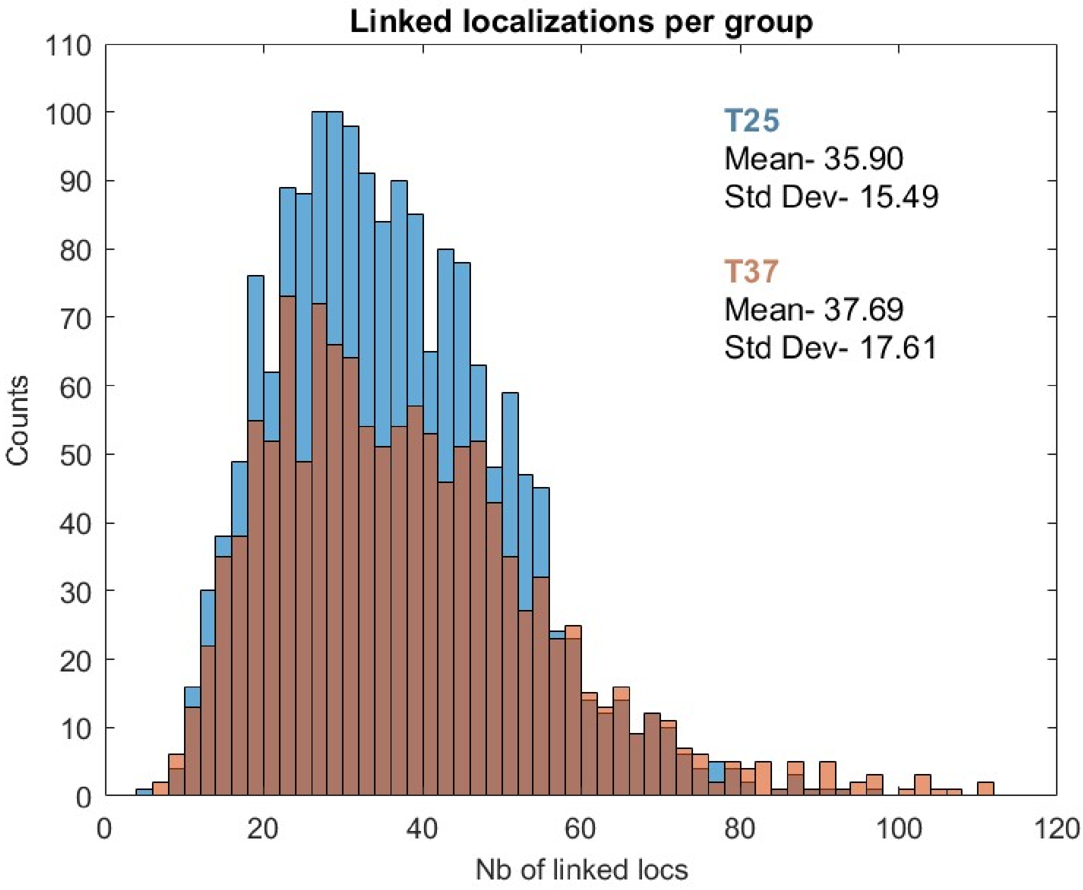

Figure S7: **25 vs 37** The graphic depicts a comparison of the linked localizations per DNA strand (500 bp) at 25 and 37°C after undergoing the analysis process (described in section VI). In the visual, group refers to an individual DNA strand.

VII. ONE DNA MOLECULE PER POLYPLEX

To ensure consistent blinking events within and outside the polyplex, we prepared control samples much diluted, where we can have on average 1 DNA strand per polyplex. The dSTORM measurements were conducted under identical conditions as those employed for other polyplex systems, at both 25 and 37°C. For the preparation of these control samples, 35.54 uL of PNIPAM/PEI microgel stock solution (11 ug/mL) and 0.1 uL of DNA (30 ug/mL) with a length of 500 bp were utilized, each appropriately diluted in MilliQ water to achieve a final volume of 50 uL. The DNA solution was introduced to the polymer solution through a simple dipping process. Additionally, 1 uL of HEPES buffer at pH 7.4 (1 M) was added to the combined solution. This mixture was placed on an oscillating tray (with a slow movement of 42 oscillations/minute) for 4 hours at room temperature. The sample was refrigerated, and measurements were conducted after 24 hours using dSTORM.

A. Image analysis for 1 DNA strand per polyplex

Subsequent analysis of the dSTORM images was performed using the Picasso software, as detailed in section V. Initially, polyplexes were localized by identifying and picking them

on the bright field image. The bright field, epifluorescence, and Render pictures were then overlaid, identifying the areas where the microgels are located. After localization, the pick function of the Render module (with a diameter of 2 px) was used to select the areas where the polyplexes were found. The data was further scrutinized using the same in-house customized Matlab routine used for other polyplex systems. Due to the high dilution, most of the microgels were empty, while few of them have from 100 to 200 localisations, which is also what we get for single DNA strands as explained in section VI and shown in Fig. S6. In addition, by comparing the consecutive frame lengths between the single DNA inside the polyplex and samples with DNA only, we have established that the DNA blinking rate is consistent both within and outside the microgel (Fig. S9).

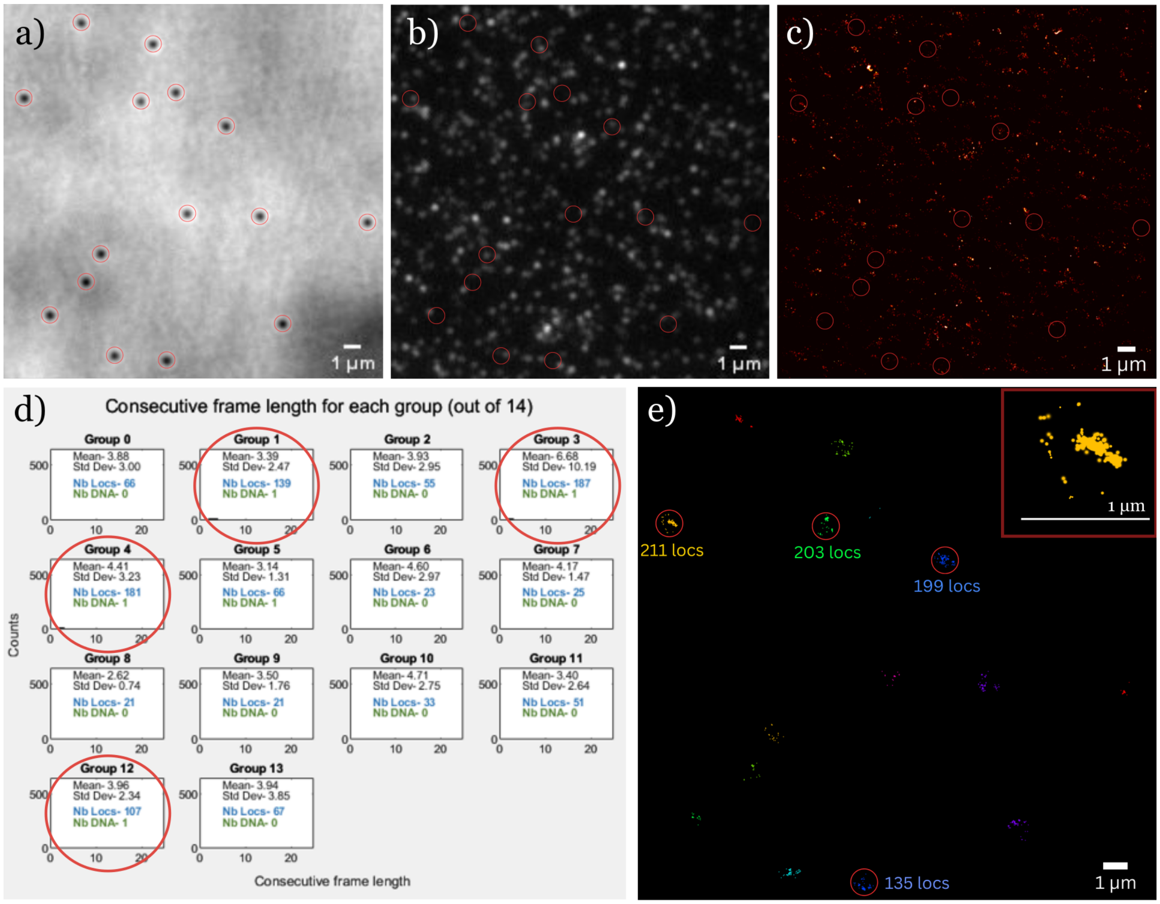

Figure S8: **1 DNA per polyplex** a) Brightfield image showing the identified and manually picked microgels. b) Epifluorescence image of the same measurement showing the picked regions where the microgels are found after overlaying the Brightfield image. c) Render image after Picasso analysis displaying DNA strands, with circles showing the regions where the microgels are situated. d) Consecutive frame length analysis of all selected groups, with many exhibiting low localizations, indicative of being empty. Polyplexes were identified based on the number of localizations, aligning with the pattern observed in the DNA-only samples. e) Render image showing picked polyplexes with a single DNA, along with the unfiltered number of localisations.Inset: zoomed image of one picked polyplex system.

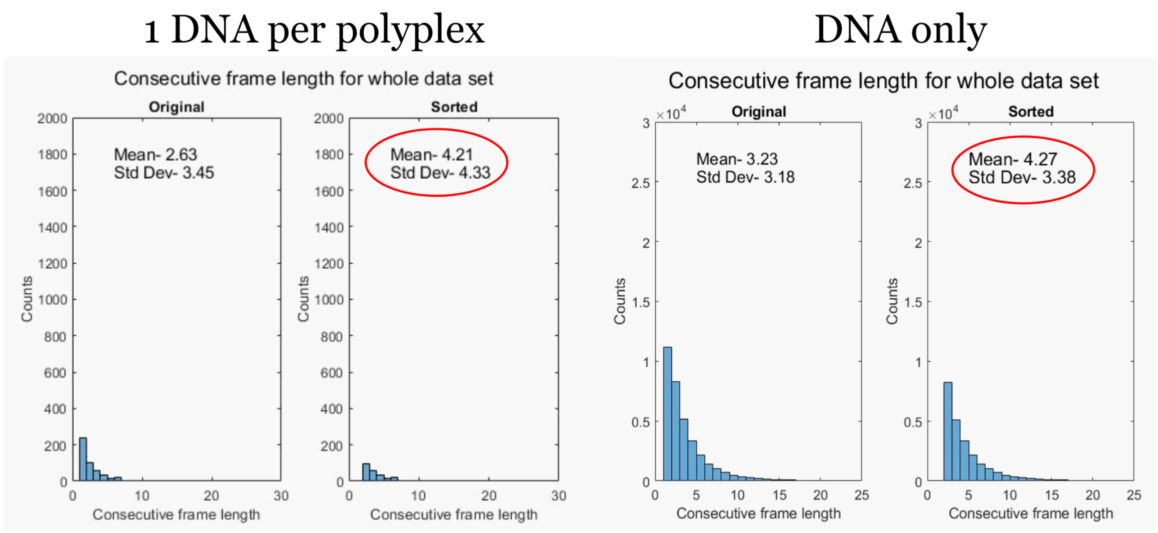

Figure S9: **Consistency in blinking behavior** The histograms depict consecutive frame lengths of the entire dataset before and after filtering for a single DNA per polyplex (on the left) and for the DNA-only dataset (on the right). Notably, both histograms exhibit comparable values, providing strong evidence that the blinking behavior remains consistent both within and outside the microgel.

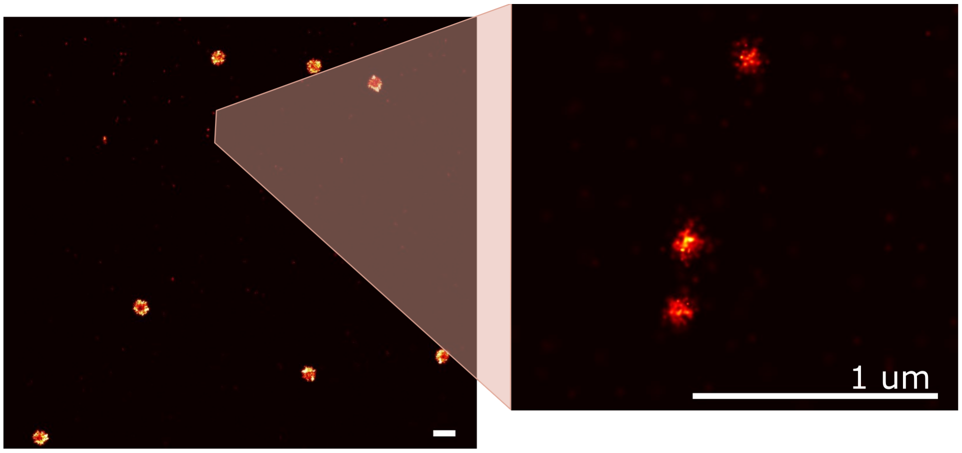

Figure S10: **Non-complexed DNA in the polyplex solution**

B. Cell Exposures and uptake of free DNA and DNA polyplexes

This study focused on single-cell imaging of macrophages (MDMs) exposed to polyplexes, utilizing ImageJ and custom scripts for analysis. Initially, the study identified cell contours to define regions of interest, isolating signals and adjusting thresholds to exclude background noise. The analysis, which concentrated on ten cells per sample, explored cell interactions with polyplexes, including cytofluorogram representations and labeling of actin, nuclei, and polyplexes. Furthermore, colocalization with nuclei was examined, and cell viability was assessed using LDH assays across various polyplex concentrations, with results summarized using statistical measures like Pearson's correlation coefficient.

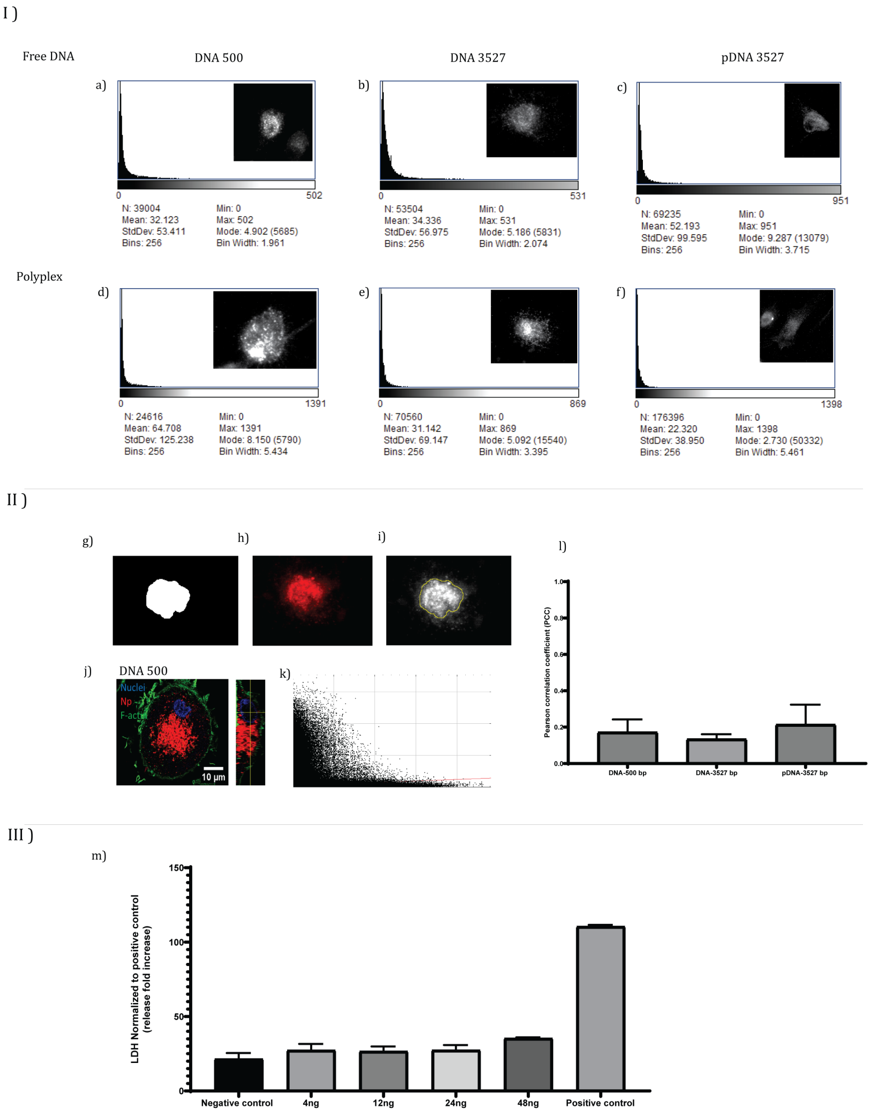

Figure S11: **Cellular uptake and colocalization** I) Representative Single-Cell Images of MDMs Exposed to Polyplexes. (a-f) MDMs exposed to Polyplexes for 6 hours followed by 36 hours post exposure and their corresponding histograms from free DNA and their corresponding polyplexes. II) representative single-cell analysis. The images were analyzed with ImageJ software using a custom script. Cell contours were manually outlined using the Alexa 647 channel to determine the region of interest (ROI) (g). Signals from free DNA and polyplexes in the Alexa 647 channel(h)were masked and an automatic threshold adjustment was applied. An inverted mask was then used to measure the raw integrated density, excluding background signals (f). This process was performed for ten individual cells per sample. (I) representative image of an MDM exposed to polyplexes. The labels correspond to F-actin (green), nuclei (blue), and polyplexes (red). Scale bar: 10 μ/m.Confocal images of single cells with the corresponding cytofluorograms. No colocalization of polyplexes with nuclei.(l) Colocalization of free DNA and representative polyplexes with the nuclei of the MDMs. The data was quantified using ImageJ software with the JACoP plugin (n = 7-10 cells). The colocalization is shown as Pearson's correlation coefficient (PCC). Whiskers represent standard deviations. III) Cell viability assessed via membrane rupture - LDH assay (m). Cell viability of MDMs macrophages after different polyplex concentrations, presented as a fold increase over positive (control). The data is the mean of the three repetitions $\pm$ standard deviationẆhiskers represent the standard deviation.

Name	Sequence	
Ori Xho fw	gactcgagcgtcgatttttgtgatgctcg	Forward primer for 3.5kb fragment of pMax-GFP
Ori Xho re	ttctcgaggtggcgaaacccgacag	Reverse primer for 3.5kb fragment of pMax-GFP

TABLE S1: **Primers for PCR labeling**

Linear DNA fragments PCR labelling	Number of thymidine residues (both strands)	Total number of bases (both strands)	Labeled nucleotides
500 bp Lamda DNA 1:1 ratio	217	1000	10,85 %
3527 bp pMax-GFP	1656	7054	11,74 %

TABLE S2: **Expected amount of incorporated label**

LabelIT reaction	Labeled nucleotides
3527 bp pMax-GFP plasmid	1,43-3,33 %

TABLE S3: **Expected amount of incorporated label (plasmid DNA**

REFERENCES

[1]B. P. Bratton, J. W. Shaevitz, and T. Abraham, "Simple experimental methods for determining the apparent focal shift in a microscope system," PLoS ONE **10** (2015), 10.1371/journal.pone.0134616.

[2]K. Mortensen and H. Flyvbjerg, ""calibration-on-the-spot": How to calibrate an emccd camera from its images," Scientific Reports **6**, 28680 (2016).
